# Still Exhausted: The Role of Residual Caregiving Fatigue on Women in Medicine and Science Across the Pipeline

**DOI:** 10.2196/47629

**Published:** 2023-06-14

**Authors:** Katherine A Meese, Laurence M Boitet, Katherine L Sweeney, Lauren Nassetta, Michael Mugavero, Bertha Hidalgo, Rebecca Reamey, David A Rogers

**Affiliations:** 1 Department of Health Services Administration University of Alabama at Birmingham Birmingham, AL United States; 2 UAB Medicine Birmingham, AL United States; 3 Department of Sociology University of Alabama at Birmingham Birmingham, AL United States; 4 Department of Pediatrics University of Alabama at Birmingham Birmingham, AL United States; 5 Department of Medicine University of Alabama at Birmingham Birmingham, AL United States; 6 Department of Epidemiology University of Alabama at Birmingham Birmingham, AL United States

**Keywords:** caregiving, women in medicine, exhaustion, childcare, burden, burnout, stress, caregiver, women, professional, child, eldercare, elderly, older adults, older adult, gerontology, family care, informal care, unpaid care, survey

## Abstract

Understanding the impact of caregiving responsibilities on women in medicine is crucial for ensuring a healthy and intact workforce, as caregiving responsibilities have the potential to affect the careers of women in health care along the entire pipeline, from students and trainees to physicians, physician-scientists, and biomedical researchers.

## Introduction

Women account for approximately 70% of the global health care workforce [1] and were disproportionately affected by the stresses of the pandemic [2]. Health care workers in all gender groups who were in caregiving roles at home, whether that be childcare or eldercare, experienced fear and anxiety about bringing the virus home [3]. However, prior research suggests that women in caregiving roles are more likely to experience stress and other adverse health impacts compared to men [4]. A recent study reported that female gender and caregiver status were predictors of increased stress during the pandemic [5]. As a result of COVID-19, female health care workers reportedly experienced a significant increase in symptoms such as depression, stress, anxiety, and insomnia [2]. Health care workers who were mothers or in mothering roles also experienced feelings of guilt during the pandemic derived from the dual expectations of worker and mother identities [3].

Even absent a pandemic, caregiving requires time and energy, which are limited resources for people with occupational demands. Family obligations can lead to physical, emotional, and financial strain on the caregiver [6]. Women working in the health care field experience increased stress and anxiety surrounding their caregiving roles at work and in the home, a phenomenon called double-duty caregiving [6]. COVID-19 only heightened these experiences for health care workers, in part due to lack of organizational support. With increases in patient volume and the closing of child and adult care facilities, women in the health care field experienced a disproportionate increase in their professional and personal caregiving and reported feeling that there was not enough support for them to care for their families from their organizations [2]. In a recent study, 49% of health care workers reported that emergency childcare needs disrupted their work, including canceled clinics and surgeries [7].

## Methods and Findings

We surveyed 6466 health care workers at a large academic medical center in the United States in 2022 regarding their stress related to caregiving, including childcare and eldercare, as well as residual stress and exhaustion from caregiving during the pandemic. Though most schools and daycares were open during this time period, the rates of exhaustion from caregiving during the pandemic remained high. We found that of all health care workers, nearly 23% (173/592) of those who identified as women indicated caregiving exhaustion from responsibilities during the pandemic, while only 14% (86/519) of those who identified as men reported this lingering stressor (*P*<.001). We also found that the perception of low organizational support and caregiving exhaustion were positively associated (*P*<.001), highlighting the weight of organizational influence on exhaustion and burnout related to caregiving.

## Discussion

Given the association of perceived organizational support with exhaustion and the well-demonstrated negative emotional, financial, and patient care–associated impacts of a burned-out workforce, health care organizations must carefully evaluate their current state and consider mechanisms for improvement. One approach to decreasing caregiving stressors is to ensure the availability of on-site childcare, which is very important to health care workers, particularly for emergency caregiving needs [7]. Other options include home-based childcare and eldercare services, concierge services and school break camps, and paid parental and sick leave, which can also be used for eldercare responsibilities. In addition to providing direct caregiving support, organizations should consider making strategic investments in employees with caregiving burdens as a recruitment and retention mechanism. For example, the Doris Duke Foundation initiated a program called the COVID-19 Fund to Retain Clinical Scientists, which invested over US $20 million in combined foundation and institutional matching funds to support clinical scientists affected by caregiving responsibilities in continuing and advancing their research agendas [8]. The money does not pay for care directly, but rather aims to aid scientists affected by caregiving responsibilities to increase their research productivity.

Many organizations have paid parental leave and paid sick time that can be used for illnesses of children, dependents, or adult family members. However, there is an important difference between having the benefit and having a culture where people feel safe and encouraged to use it. Others have demonstrated that organizational culture is a correlate of parental leave use [9-11]. Organizations must observe and measure the use of these benefits and rectify any cultural practices or norms that dissuade people from using them. Doing so is not only necessary to help alleviate the accrual of caregiving fatigue but is also important for recruitment and retention efforts [11].

## Conclusion

These findings highlight the need for organizations to prioritize support for those affected by caregiving responsibilities through the lens of gender-specific crisis response. Especially in the context of the evolving pandemic, it is important to remember that in addition to increased non–work-related caregiving responsibilities, women health care workers are predisposed to increased risk of disease exposure and face barriers to resources, heavier workloads, and decreased leadership and decision-making opportunities [12-17]. This is especially salient when considering the recent increased exodus of trainees from training programs, with trainees citing lack of support for working parents [18] and negative effects of increased caregiving responsibilities on productivity [19]. When job demands exceed the resources available for the trainees and workforce to perform optimally, organizations risk the loss of current and future employees [20].

The lingering effect of caregiver exhaustion from the pandemic highlights the importance of caregiver support as a crisis management and emergency preparedness priority. Though the current stress of caregiving in the endemic stages of COVID-19 may be low, it is evident that health care workers remain deeply affected by the challenges of this time period [21]. Organizational leaders must keep in mind that the reopening of schools and care facilities does not resolve the lingering impact on the women who cared for dependents during the years of the pandemic.
